# Dietary selenium intake based on the Chinese Food Pagoda: the influence of dietary patterns on selenium intake

**DOI:** 10.1186/s12937-018-0358-6

**Published:** 2018-05-09

**Authors:** Jing Wang, Linsheng Yang, Hairong Li, Yonghua Li, Binggan Wei

**Affiliations:** 1 Key Laboratory of Land Surface Pattern and Simulation, Institute of Geographical Sciences and Natural Resources Research, Chinese Academy of Sciences, 11 A Datun Road, Beijing, 100101 People’s Republic of China; 2 College of Resources and Environment, University of Chinese Academy of Sciences, Beijing, 100049 People’s Republic of China

**Keywords:** Selenium, Dietary intake, Chinese Food Pagoda, China Total Diet Study

## Abstract

**Background:**

Selenium (Se) is essential for humans, with many critical roles in physiological and pathophysiological processes. Fish, eggs and meats are usually the rich food sources of Se. To improve the nutritional status of population, a new version of balanced dietary pattern in the form of the Chinese Food Pagoda (2016) was proclaimed. This study aimed to evaluate the contribution of this balanced dietary pattern to daily Se intake, and to assess Se intake status of Chinese residents under this Food Pagoda scenario.

**Methods:**

Based on the food consumption recommended in the Food Pagoda, this study collected the data of Se contents in various food composites and estimated dietary Se intakes (EI_TDS_) in 12 provinces from the 4th China Total Diet Study. The estimated Se intakes based on the Chinese Food Pagoda (EI_CHFP_) in 12 provinces were calculated. EI_TDS_ and EI_CHFP_ in various food groups among different regions were compared.

**Results:**

The average EI_CHFP_ in all regions, within the range of 66.23–145.20 μg/day, was greater than the China recommended nutrient intake (RNI) (60 μg/day). None of the highest EI_CHFP_ went beyond the tolerable upper intake level of Se (400 μg/day). Animal source foods should be the primary source of daily Se intake according to the EI_CHFP_. The average EI_TDS_ in China (88 μg/day) was in line with its range of EI_CHFP_ (81.01–124.25 μg/day), but that in half of the regions failed to achieve their lowest EI_CHFP_. Significant differences between EI_TDS_ and EI_CHFP_ were observed in cereal food, aquatic and dairy products (*P* < 0.05), among which Se intake from aquatic and dairy products presented seriously insufficient in almost all regions.

**Conclusions:**

The ideal dietary pattern recommended in the Food Pagoda can meet the daily requirements of Chinese population for Se intake to maintain optimal health. From the perspective of the balanced diet and Se-rich sources, the consumption of aquatic products should be increased appropriately to improve the general Se intake level of Chinese population.

## Background

Selenium (Se) is an essential micronutrient for human health, with critical roles in redox homeostasis, antioxidant defense and immune system [1, 2]. Insufficient or excessive Se intakes are linked to many acute and chronic diseases [3–8]. In particular, problems related to Se deficiency are an emerging issue for human health worldwide [9]. It is estimated that 15% of the global population suffers Se deficiency of different degrees [10]. China as one of the 40 Se-deficient countries has over 105 million people facing adverse health impacts due to Se deficiency [11, 12]. Owing to large variations in food Se, the dietary Se intake varies considerably among regions, normally being consistent with Se distribution in the environment. In China, low Se intakes are primarily found in the low-Se geographic belt from northeast to southwest, with a mean of 27.6 μg/day; while high Se intakes are observed in Se-rich areas, with a mean of 85.5 μg/day; in some selenosis areas, the Se intake can even reach up to 1253.7 μg/day on average [12]. Considering its narrow range between the necessary and the toxic dose, an optimal daily Se intake is required to maintain public health.

**Fig. 1:**
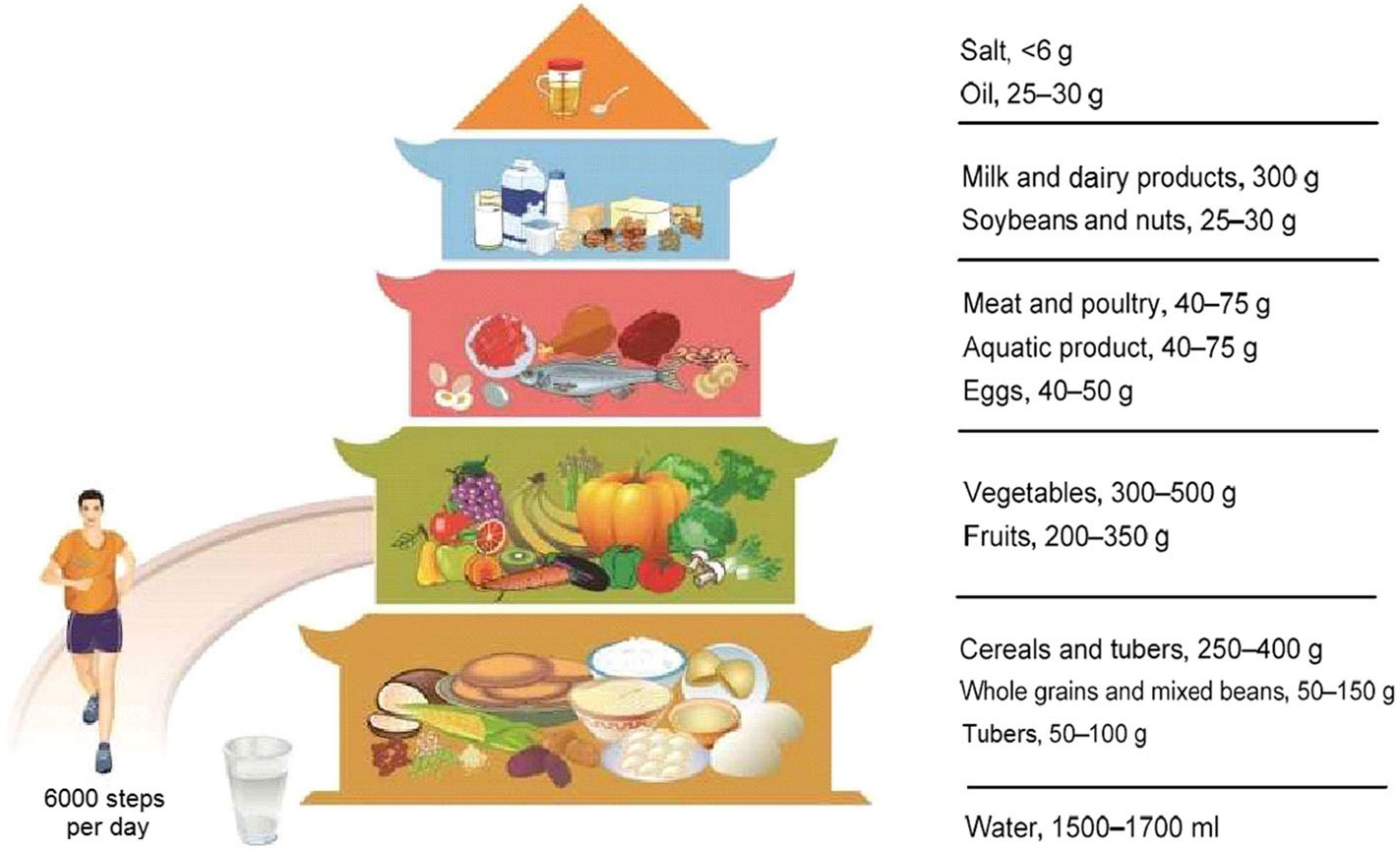
Food Guide Pagoda for Chinese residents [16]

A reasonable diet is the crucial determinant for daily Se intake [13]. It is well known that cereals, fish, eggs and meats are the major dietary sources of Se [12, 14, 15]. In response to stronger demands for healthy growth of people, the Chinese government proclaimed a new version of the Dietary Guidelines for Chinese Residents in the form of the Food Pagoda (Fig. 1), based on principles of nutritional science and the current national situation [16]. Five levels of the recommended consumption corresponding to five food groups are involved in the Food Pagoda, covering the essential foods we should consume in daily life [17]. This Pagoda recommends a relatively ideal dietary pattern to improve the general nutrition of Chinese residents. However, whether it can meet the daily requirements of Se intake for general population and achieve the optimal daily Se intake has yet to be ascertained.

In the past decades of China, Se deficiency diseases, for e.g. Kashin-Beck disease and Keshan disease, have been prevalent in low-Se areas, with particularly high morbidity in underdeveloped regions. Apart from low Se contents in local foods, unreasonable food consumption patterns were also considered as one of the main reasons for deficient Se intake [18]. A study on dietary Se intake in 1990s found that urban residents consumed more Se-rich foods such as meats, seafood, eggs and dairy products than rural residents, resulting in contrasting Se intakes between the two populations [18]. With the rapid growth of China’s economy, food supply and diversity increased dramatically [19]. Since the balanced diet conforming to the Chinese Food Pagoda is deemed as an ideal dietary pattern to promote nutrition, it is necessary to assess the Se intake level under the scenario of this Food Pagoda, and to be clear about the gap between this level and the current Se intake status of Chinese population in different provinces. This is the first study to evaluate the Se intake of Chinese residents associated with the 2016 Chinese Food Pagoda and discuss the influence of dietary patterns on Se intake. It will be valuable for the future research on daily optimal Se intake and also for the government to put forward proper strategies on Se supplementation. Therefore, this study aimed to: 1) test whether compliance with the Food Pagoda could meet daily requirements of Se intake for Chinese residents; 2) make quantitative comparisons of the China Total Diet Study-based estimates of Se intake (EI_TDS_) with the Food Pagoda-based estimates of Se intake (EI_CHFP_) in different food groups.

## Methods

### Data source

The China TDS is a national survey to investigate the levels of various nutrients and chemical contaminants in foods and assess their dietary exposure for the Chinese population [20]. The data of food Se contents and the EI_TDS_ in China and 12 provinces used in this study were directly obtained from results of the 4th China TDS in 2007 [21]. Hereinto, the analysis of food Se was conducted by the National Institute of Nutrition and Food Safety. EI_TDS_ was calculated by multiplying determined food Se contents with the investigated food consumption data.

### Food consumption survey

The 4th China TDS was carried out in 2007, with a similar design and experimental methods to the 3rd China TDS in 1990 [22]. The Chinese Centre for Disease Control and Prevention organized the food consumption survey. Multistage random cluster sampling method and food composites approach were used in this survey. A total of 12 provinces were selected to represent the average dietary patterns of different areas of China, covering about 50% of the total Chinese population. These provinces consist of Heilongjiang (HLJ), Liaoning (LN), Hebei (HeB), Shaanxi (ShX), Ningxia (NX), Henan (HN), Shanghai (ShH), Fujian (FJ), Jiangxi (JX), Guangxi (GX), Hubei (HuB), and Sichuan (SC). Three survey sites (two rural counties and one urban city) were randomly selected in each province as food sampling sites, and 30 households were sampled randomly from each site. 1080 households in total were covered in the survey. The food consumption pattern in each province was determined by a 3-day household dietary survey (including weighing and recording) and 24-h recalls. The average daily consumption of each food category by a standard Chinese adult man (aged 18–45, 63 kg body weight, light physical activity) was used as the standard food consumption pattern, and was calculated from the total household food consumption [22].

### Samples collection and analysis

Food samples were collected from local food markets, grocery stores and rural households in each survey site. All food items were aggregated into 12 groups, including cereals, beans, tubers, meat and poultry, eggs, aquatic products, milk and dairy products, vegetables, fruits, sugars, water and beverages, and alcohol. These samples were cooked and prepared according to the local habits, and then blended to form composites with weights proportional to the average daily consumption for the province [21]. The prepared food composites were shipped to the National Institute of Nutrition and Food Safety for analysis [21]. Total Se content in food composites was determined by the inductively coupled plasma mass spectrometry (Agilent 7500a ICP-MS) after microwave digestion of 0.3–0.5 g (solid) or 4–5 mL (liquid) in a mixture of 6 mL of concentrated HNO_3_ and 2 mL of 30% H_2_O_2_. Reagent blank, standard reference materials, and parallel samples were determined simultaneously to maintain the reliability of analysis. Limit of detection for Se was defined as three-times of the standard deviation of baseline value [21].

### Calculation of dietary se intake based on the Chinese food pagoda (EI_CHFP_)

The ranges of EI_CHFP_ in China and 12 provinces were calculated according to the following equation [20]:

$$ {\mathrm{EI}}_{\mathrm{CHFP}}=\mathrm{C}\times \mathrm{m}, $$

where C (μg/g) is the concentration of Se in each food group determined in the 4th China TDS, including 12 food groups in 12 provinces (as listed in Table 1); m (g/d) is the consumption of corresponding food groups recommended in the Dietary Guidelines and Food Pagoda for Chinese Residents (2016) (as shown in Fig. 1). The lower and upper limits of recommended consumption were used for calculating the lowest and highest EI_CHFP_, respectively. In terms of Se contents in food groups, the lowest values in staple food like cereals, beans and tubers were found in Hubei, Liaoning and Heilongjiang province, which was broadly consistent with the distribution of low-Se belt in China [23]. It can thus be confirmed that food Se contents determined in the TDS are reliable.

**Table 1:** Concentrations of Se in various food groups in China and 12 provinces (μg/g)^a^

Food Group	HLJ	LN	HeB	ShX	HN	NX	ShH	FJ	JX	HuB	SC	GX	AVG
Cereals	0.004	0.003	0.028	0.062	0.010	0.036	0.013	0.008	0.031	0.003	0.020	0.016	0.020
Beans	0.027	0.022	0.022	0.048	0.023	0.017	0.012	0.020	0.011	0.028	0.043	0.053	0.027
Tubers	0.027	0.024	0.023	0.063	0.015	0.058	0.006	0.011	0.008	0.002	0.017	0.048	0.025
Meat	0.111	0.157	0.121	0.166	0.238	0.173	0.219	0.124	0.210	0.186	0.205	0.276	0.182
Eggs	0.172	0.146	0.338	0.549	0.266	0.120	0.291	0.276	0.164	0.285	0.375	0.249	0.269
Aquatic product	0.135	0.708	0.591	0.366	0.456	0.121	0.462	0.367	0.468	0.231	0.422	0.597	0.410
Dairy product	0.072	0.022	0.022	0.018	0.023	0.038	0.028	0.015	0.155	0.202	0.019	0.048	0.055
Vegetables	0.035	0.012	0.065	0.146	0.110	0.042	0.079	0.079	0.020	0.068	0.041	0.089	0.066
Fruits	0.002	0.002	0.002	0.002	0.022	0.008	0.002	0.002	0.002	0.002	0.002	0.025	0.006
Sugars	0.032	0.002	0.118	0.011	0.002	0.047	0.002	0.023	0.002	0.002	0.002	0.101	0.029
Water	0.002	0.005	0.002	0.002	0.002	0.004	0.002	0.002	0.002	0.002	0.002	0.002	0.002
Alcohol	0.006	0.005	0.002	0.002	0.002	0.002	0.009	0.008	0.002	0.004	0.002	0.008	0.004

Abbreviations: *HLJ* Heilongjiang, *LN* Liaoning, *HeB* Hebei, *ShX* Shaanxi, *NX* Ningxia, *HN* Henan, *ShH* Shanghai, *FJ* Fujian, *JX* Jiangxi, *GX* Guangxi, *HuB* Hubei, *SC* Sichuan, *AVG* average

^a^All the data of Se contents in different food groups were from the 4th China TDS [21]

### Statistical analysis

Data processing and chart production were mainly performed with the Microsoft Office Excel 2013 and SPSS 23.0. Coefficients of variation (CV) were calculated for the average EI_TDS_ and EI_CHFP_ in each food group. *T* test was used when comparing the difference between the average EI_TDS_ and EI_CHFP_ in various food categories.

## Results

### EI_CHFP_ in China and different regions

**Fig. 2:**
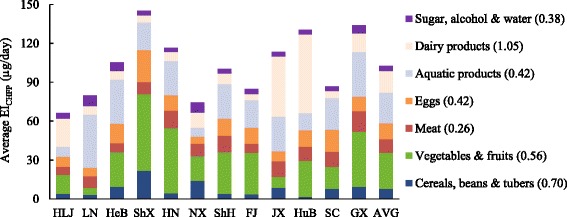
The average EI_CHFP_ in different food groups in 12 provinces and China^a^ Abbreviations: *HLJ* Heilongjiang, *LN* Liaoning, *HeB* Hebei, *ShX* Shaanxi, *NX* Ningxia, *HN* Henan, *ShH* Shanghai, *FJ* Fujian, *JX* Jiangxi, *GX* Guangxi, *HuB* Hubei, *SC* Sichuan, *AVG* average, *EI*_*CHFP*_ estimated Se intake based on the Chinese Food Pagoda. ^a^Numbers in parentheses are coefficients of variation in each food group; the same as below

Based on the concentrations of food Se in Table 1 and the food consumptions recommended in the Food Pagoda, results of the EI_CHFP_ were shown in Table 2. It was observed that the average EI_CHFP_ in 12 provinces were all greater than the China recommended nutrient intake (RNI) of Se (60 μg/day). The lowest EI_CHFP_ was also higher than the RNI in almost all regions with the exception of Heilongjiang and Ningxia province which might be related to the relatively low Se levels in their local food (Table 1). None of the highest EI_CHFP_ went beyond the tolerable upper intake level of Se (400 μg/day) set by the Chinese Nutrition Society [24]. Owing to the variation of Se levels in the local food, the average EI_CHFP_ varied greatly among regions, ranging from 66.23 to 145.20 μg/day. The highest average EI_CHFP_ was observed in Shaanxi province where Se levels in staple food and vegetables were the highest; while the lowest was found in Heilongjiang province where Se contents in all kinds of food groups were relatively low. It could be seen from Fig. 2 that differences of the average EI_CHFP_ among regions mainly lay in dairy products (CV = 1.05), cereals, beans and tubers (CV = 0.70), as well as vegetables and fruits (CV = 0.56). Animal source foods including meat, eggs, aquatic and dairy products made the highest contribution to daily Se intake in all regions according to the Food Pagoda eating patterns, ranging from 41.8 to 81.9%.

**Table 2:** The average EI_CHFP_ and its ranges in China and different provinces (μg/day)

Regions	The average EI_CHFP_^a^	The lowest EI_CHFP_	The highest EI_CHFP_
HLJ	66.23	55.21	77.25
LN	79.75	60.90	98.60
HeB	105.32	79.39	131.25
ShX	145.20	112.65	177.75
HN	116.59	89.04	144.15
NX	74.41	58.92	89.90
ShH	100.32	77.59	123.05
FJ	84.81	65.05	104.58
JX	113.46	96.47	130.45
HuB	130.41	113.89	146.93
SC	86.75	67.42	106.08
GX	133.94	101.41	166.48
AVG	102.63	81.01	124.25

Abbreviations: *HLJ* Heilongjiang, *LN* Liaoning, *HeB* Hebei, *ShX* Shaanxi, *NX* Ningxia, *HN* Henan, *ShH* Shanghai, *FJ* Fujian, *JX* Jiangxi, *GX* Guangxi, *HuB* Hubei, *SC* Sichuan, *AVG* average, *EI*_*CHFP*_ estimated Se intake based on the Chinese Food Pagoda

^a^The average EI_CHFP_ = (the lowest EI_CHFP_ + the highest EI_CHFP_)/2

### EI_TDS_ in China and different regions

**Fig. 3:**
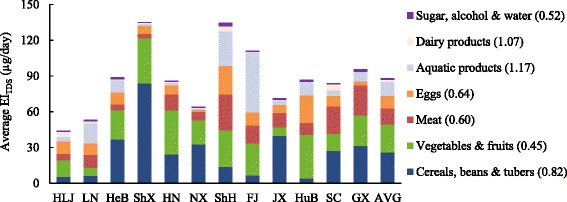
The average EI_TDS_ in different food groups in 12 provinces and China Abbreviations: *HLJ* Heilongjiang, *LN* Liaoning, *HeB* Hebei, *ShX* Shaanxi, *NX* Ningxia, *HN* Henan, *ShH* Shanghai, *FJ* Fujian, *JX* Jiangxi, *GX* Guangxi, *HuB* Hubei, *SC* Sichuan, *AVG* average, *EI*_*TDS*_ estimated Se intake based on the China Total Diet Study.

According to the survey data from the 4th China TDS, the EI_TDS_ of daily dietary Se in various food groups in 12 provinces were presented in Fig. 3. Generally, the level of average EI_TDS_ for Chinese adults (88 μg/day) was slightly higher than the China RNI of Se (60 μg/day). Large geographical variation of dietary Se intake was observed among regions in China. As shown in Fig. 3, the highest EI_TDS_ was found in Shaanxi province (135.31 μg/day) at 2.3 times of RNI, followed by Shanghai (134.58 μg/day); while the lowest EI_TDS_ was observed in Heilongjiang province (43.86 μg/day), followed by Liaoning (53.35 μg/day), which were the only two provinces below the RNI. The EI_TDS_ in most of the provinces, such as Hebei, Henan, Jiangxi, Hubei, Sichuan and Guangxi, was within the range of 71.5–95.72 μg/day. Great variations of EI_TDS_ in food groups among regions were observed in aquatic products (CV = 1.17), dairy products (CV = 1.07), and cereals, beans and tubers (CV = 0.82), making differences in the major contributors to dietary Se intake in 12 provinces. Animal source food was found making the highest contribution to dietary Se intake in more than half of the regions, including Heilongjiang, Liaoning, Shanghai, Fujian, Hubei, Sichuan, and Guangxi province ranging from 38.1 to 69.4%. Particularly, aquatic products corresponded to the highest contributors in Fujian (45.4%) and Liaoning (30.9%) province. By contrast, cereals and tubers contributed the most to daily dietary Se intake in Shaanxi, Hebei, Ningxia, and Jiangxi province within the range of 41.6–61.9%. Vegetables made a predominant contribution to daily Se intake in Henan and Hubei province, accounting for 41.2 and 41.7% of the total intake respectively. Despite all this, it can be observed that animal foods and cereals are still the major sources of daily dietary Se intake in most regions of China, which is similar to previous studies in China [25].

### Comparison of EI_TDS_ with EI_CHFP_

**Fig. 4:**
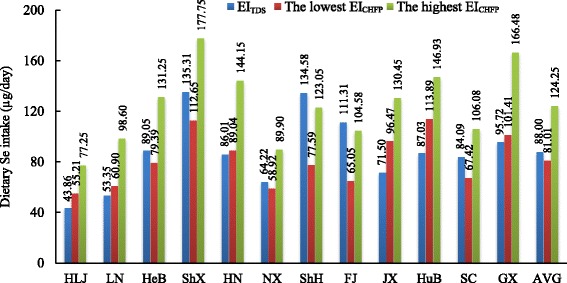
Comparison of daily dietary Se between EI_TDS_ and EI_CHFP_ in 12 provinces in China Abbreviations: *HLJ* Heilongjiang, *LN* Liaoning, *HeB* Hebei, *ShX* Shaanxi, *NX* Ningxia, *HN* Henan, *ShH* Shanghai, *FJ* Fujian, *JX* Jiangxi, *GX* Guangxi, *HuB* Hubei, *SC* Sichuan, *AVG* average, *EI*_*TDS*_ estimated Se intake based on the China Total Diet Study, *EI*_*CHFP*_ estimated Se intake based on the Chinese Food Pagoda.

Results of the comparison between total EI_TDS_ and EI_CHFP_ were depicted in Fig. 4. In terms of the whole country, the average EI_TDS_ (88 μg/day) just fell into the range of its EI_CHFP_ (81.01–124.25 μg/day). This indicated that the daily dietary Se intake of Chinese population was overall in line with the recommendation proposed by the Chinese Food Pagoda. However, similar situation was only found in Hebei, Shaanxi, Ningxia, and Sichuan province. The majority of regions including Heilongjiang, Liaoning, Henan, Jiangxi, Hubei, and Guangxi province had a lower EI_TDS_ when compared with their corresponding lowest EI_CHFP_. The gap between them ranged from 3.03 μg/day to 26.86 μg/day, with relatively bigger gaps in Hubei (26.86 μg/day) and Jiangxi (24.97 μg/day) province. By contrast, the EI_TDS_ in Shanghai and Fujian province (134.58 and 111.31 μg/day) were even higher than their highest EI_CHFP_ (123.05 and 104.58 μg/day). This comparison was just between the EI_TDS_ and EI_CHFP_ which were calculated under different food consumption patterns, regardless of the China RNI.

The EI_TDS_ and their corresponding ranges of EI_CHFP_ from each food category among different regions were calculated and integrated according to the five levels of food groups classified by the Food Pagoda. As listed in Table 3, EI_TDS_ from the first level of the Food Pagoda (cereals, tubers and beans) in 12 provinces all exceeded their recommended ranges; while those from the fourth level (dairy products) were all far below their recommended amounts. This situation was more remarkable in Shaanxi, Hebei, Henan and Jiangxi province where the EI_TDS_ from cereals, tubers and beans were more than 3 times of their highest EI_CHFP_, while those from dairy products were substantially poor. Additionally, Se intake from the third level (meat, eggs and aquatic products) varied greatly among regions, where only Heilongjiang and Hubei province had appropriate Se intake from this level. Those in Shanghai and Fujian province were much higher than their upper limits of EI_CHFP_, which might account for their higher EI_TDS_ in Fig. 4. Almost all provinces had adequate Se intake from the second level of the Food Pagoda (vegetables and fruits) with the exception of Shaanxi, Henan and Guangxi province being slightly lower than their recommendations.

**Table 3:** EI_TDS_ and EI_CHFP_ in different food categories in 12 provinces of China (μg/day)^a^

Food category	Cereals, tubers and beans		Vegetables & Fruits		Meat, eggs and aquatic products		Dairy products	
	EI_TDS_	EI_CHFP_ ranges	EI_TDS_	EI_CHFP_ ranges	EI_TDS_	EI_CHFP_ ranges	EI_TDS_	EI_CHFP_
HLJ	5.55	2.83–5.25	13.50	10.90–18.20	19.96	16.72–27.05	4.09	21.60
LN	6.16	2.35–4.40	6.98	4.00–6.70	37.01	40.44–72.18	2.04	6.60
HeB	37.02	7.30–11.80	24.17	19.90–33.20	25.37	42.00–70.30	0.57	6.60
ShX	83.70	16.75–27.30	38.19	44.20–73.70	11.99	43.24–67.35	0.86	5.40
HN	24.41	3.33–5.65	36.72	37.40–62.70	23.30	38.40–65.35	0.73	6.90
NX	32.66	10.53–17.45	20.10	14.20–23.80	10.19	16.56–28.05	0.58	11.40
ShH	14.01	3.20–5.10	30.54	24.10–40.20	82.92	38.88–65.63	4.05	8.40
FJ	6.57	2.65–4.50	27.05	24.10–40.20	76.30	30.68–50.63	0.48	4.50
JX	39.61	6.88–10.65	7.48	6.40–10.70	23.16	33.68–59.05	0.05	46.50
HuB	4.31	1.40–2.50	36.43	20.80–34.70	43.99	28.08–45.53	0.22	60.60
SC	27.19	5.93–9.85	14.21	12.70–21.20	36.68	40.08–65.78	4.79	5.70
GX	31.35	6.93–12.25	25.77	31.70–53.25	36.48	44.88–77.93	0.22	14.40
AVG	26.05	5.93–9.85	23.43	21.00–35.10	35.61	34.44–57.85	1.56	16.50

Abbreviations: *HLJ* Heilongjiang, *LN* Liaoning, *HeB* Hebei, *ShX* Shaanxi, *NX* Ningxia, *HN* Henan, *ShH* Shanghai, *FJ* Fujian, *JX* Jiangxi, *GX* Guangxi, *HuB* Hubei, *SC* Sichuan, *AVG* average, *EI*_*TDS*_ estimated Se intake based on the China Total Diet Study, *EI*_*CHFP*_ estimated Se intake based on the Chinese Food Pagoda

^a^The data of EI_TDS_ in different food categories were from the 4th China TDS [21]

## Discussion

Compared with other countries in the world, the dietary Se intake of Chinese population (88 μg/day) was relatively moderate. It was higher than numerous countries throughout Europe (including the UK) and the Middle East where the reported daily Se intake were less than 55 μg/day [26], but lower than those from certain developed countries, such as the US where 133.5 μg/day of average Se intake for men was reported [27]. However, great geographical variations of Se intake still existed in China. There are many possible factors that can have an effect on Se intake and result in these variations. These factors primarily include disparities in dietary patterns, consumption habits, food culture, economic levels, and also Se contents in food among different regions [18, 28, 29]. It is difficult to distinguish which factor plays a predominant role in dietary Se intake since the situation varies from regions to regions. For example, cereals and tubers made the highest contribution to daily dietary Se intake in Shaanxi, Hebei, Ningxia, and Jiangxi province, accounting for 41.6–61.9%; while 45.4 and 30.9% of daily Se intake were from aquatic products in Fujian and Liaoning province, making the highest contributors. The former is presumably ascribed to the relatively high Se levels in local crops (Table 1), as well as the food culture where tend to have large consumptions of flour or rice [21]; the latter, however, may be attributed to the consumption habits developed by their water-adjacent living environment. Correlations between Se contents and EI_TDS_ in each food group also showed that highly significant correlations were only observed in foods with large consumptions, including cereals (*r* = 0.936, *P* < 0.01), tubers (*r* = 0.787, *P* < 0.01), vegetables (*r* = 0.895, *P* < 0.01) and fruits (*r* = 0.769, *P* < 0.01). Foods with relatively high Se contents but small consumptions, such as seafood, eggs, and meats, presented poor correlations with Se intake (*P* > 0.05). In this study, the influence of dietary patterns on Se intake was discussed separately by quantitative comparison between EI_TDS_ and EI_CHFP_ which were calculated using the same food Se contents [21].

In the first place, the calculated results of EI_CHFP_ being greater than the China RNI (Table 2) has well suggested that Se intake complying with the balanced dietary patterns recommended in the Chinese Food Pagoda could meet the daily requirements of the majorities in China, even though Se contents in various food groups vary greatly among regions. By comparison with the total EI_TDS_, it was found that half of regions failed to meet the minimum Se intake requirements of the Food Pagoda, indicating that dietary patterns in these regions may exist irrationality. When compared within various food categories, it was noteworthy that significant differences between the average EI_CHFP_ and EI_TDS_ were found in cereals, tubers and beans (*t* = − 2.975, *P* = 0.007), aquatic products (*t* = 2.468, *P* = 0.021) and dairy products (*t* = 3.089, *P* = 0.005). According to the average EI_CHFP_ in the whole country, there should be 7.7% of Se intake contributed from staple food, 23.0 and 16.1% contributed from aquatic and dairy products. Clearly, the current Se intake from staple food has been sufficient, accounting for 29.6% of the average EI_TDS_ in China; while that from aquatic and dairy products is largely deficient across the country, only accounting for 13.5 and 1.8% of the average EI_TDS_ in China. In fact, Chinese population consumed very limited milk and dairy products every day, with an average of 28.4 g/day per capita according to the food consumption data in the 4th China TDS [21], far below 300 g/day per capita recommended in the Pagoda. The consumption of aquatic products (29.0 g/day per capita) was also less than the lower limit of the recommended amount (40 g/day per capita) [16, 21]. However, compared with other animal source foods, milk and dairy products are not very good sources for Se. The average Se content in dairy products was only 0.055 μg/g, far less than that in aquatic products (0.410 μg/g) [21]. Therefore, from the view of the balanced dietary pattern and Se sources, Se nutrition for the general population can be further improved by properly increasing the consumption of seafood and its by-products.

It is well known that human Se intake is closely associated with Se levels in foodstuffs and dietary pattern. The former is strongly dependent on the Se in soil which varies significantly across different regions of world [30]; the latter, however, can be changed with the increase of food supply and diversity as well as dietary habits. In the present study, for instance, in Heilongjiang and Liaoning province where EI_TDS_ has not reached the China RNI, their Se intake can be further improved by slightly adjusting eating patterns on the basis of Food Pagoda, such as consuming more fish, milk and dairy products, and so on. Even in some regions where Se intake has achieved the RNI, it can still be supplemented by adjusting the consumption of Se-rich foods within the recommended ranges to obtain Se-associated health benefit. The present study underestimated the influence of soil Se distribution on Se intake, because even though Se levels in food is a reflection of soil Se in most instances, soil Se contents do not help in cases where the food locally produced is sold and consumed by residents in other regions. Instead, by calculating the contribution of the balanced diet to daily Se intake, this study demonstrated that Se intake complying with balanced dietary patterns can achieve or even exceed the China RNI in different regions no matter how greatly their Se contents in food vary. Thus, it is believed that the daily requirement of the general population for Se can be satisfied if the Chinese Dietary Guidelines and the Food Guide Pagoda are strictly obeyed.

## Conclusions

Attempts can be made to improve the general Se intake levels by adjusting eating patterns. The present study made it clear that the balanced dietary pattern based on the Chinese Dietary Guidelines and Food Pagoda could meet daily requirements of the majorities for Se in China under the current Se levels in food. However, the comparison between EI_TDS_ and EI_CHFP_ showed that Se intake in half of the regions could not achieve their lowest EI_CHFP_. The differences between them among regions mainly lay in cereal food, aquatic and dairy products. Se intake from staple food for Chinese population may have been sufficient, and more Se nutrition can be taken from aquatic products in terms of a well-balanced diet.

## Abbreviations

AVG: average
CV: coefficient of variation
EI_CHFP_: Estimated Se intake based on the Chinese Food Pagoda
EI_TDS_: Estimated Se intake based on the China Total Diet Study
FJ: Fujian
GX: Guangxi
HB: Hebei
HLJ: Heilongjiang
HN: Henan
HuB: Hubei
ICP-MS: inductively coupled plasma mass spectrometry
JX: Jiangxi
LN: Liaoning
NX: Ningxia
RNI: Recommended nutrient intake
SC: Sichuan
ShH: Shanghai
ShX: Shaanxi
TDS: Total diet study

## References

[1] Rayman MP (2012). Selenium and human health. Lancet.

[2] Kryukov GV, Castellano S, Novoselov SV, Lobanov AV, Zehtab O, Guigó R (2003). Characterization of mammalian selenoproteomes. Science.

[3] Fairweather-Tait SJ, Bao YP, Broadley MR, Collings R, Ford D, Hesketh JE (2011). Selenium in human health and disease. Antioxid Redox Sign.

[4] Méplan C, Hesketh J (2014). Selenium and cancer: a story that should not be forgotten-insights from genomics. Cancer Treat Res.

[5] Guo X, Ma WJ, Zhang F, Ren FL, Qu CJ, Lammi MJ (2014). Recent advances in the research of an endemic osteochondropathy in China: Kashin-Beck disease. Osteoarthr Cartilage.

[6] Harthill M (2011). Review: micronutrient selenium deficiency influences evolution of some viral infectious diseases. Biol Trace Elem Res.

[7] Wang XL, Yang TB, Wei J, Lei GH, Zeng C (2016). Association between serum selenium level and type 2 diabetes mellitus: a non-linear dose-response meta-analysis of observational studies. Nutr J.

[8] Manzanares W, Hardy G (2016). Can dietary selenium intake increase the risk of toxicity in healthy children?. Nutrition.

[9] Reis ARD, EI-Ramady H, Santos EF, Gratão PL, Schomburg L. Overview of selenium deficiency and toxicity worldwide: affected areas, selenium-related health issues, and case studies. In: Pilon-Smits E, Winkel L, Lin ZQ (eds) selenium in plants. Plant Ecophysiology 2017. 11:209–30.

[10] Tan LC, Nancharaiah YV, van Hullebusch ED, Lens PNL (2016). Selenium: environmental significance, pollution, and biological treatment technologies. Biotechnol Adv.

[11] Xu ZC, Shao HF, Li S, Zheng C (2012). Relationships between the selenium content in flue-cured tobacco leaves and the selenium content in soil in Enshi, China tobacco-growing area. Pak J Bot.

[12] Dinh QT, Cui ZW, Huang J, Tran TAT, Wang D, Yang WX (2018). Selenium distribution in the Chinese environment and its relationship with human health: a review. Environ Int.

[13] Yu GH, Wen YM, He SY, Zhang L, Dong HY (2007). Food selenium content and resident daily selenium intake in Guangzhou City. Chin J Appl Ecol.

[14] Santos MD, Júnior FMRDS, Muccillo-Baisch AL (2017). Selenium content of Brazilian foods: a review of the literature values. J Food Compos Anal.

[15] Choi Y, Kim J, Lee HS, Kim C, Hwang IK, Park HK (2009). Selenium content in representative Korean foods. J Food Compos Anal.

[16] The Chinese Nutrition Society. The Food Guide Pagoda for Chinese Residents. 2016. http://dg.cnsoc.org/upload/images/source/20160519163856103.jpg. Accessed 20 June 2016.

[17] Wang SS, Lay S, Yu HN, Shen SR (2016). Dietary guidelines for Chinese residents (2016): comments and comparisons. J Zhejiang Univ-Sci B (Biomed & Biotechnol).

[18] Zhang ZW, Shimbo S, Qu JB, Watanabe T, Nakatsuka H, Matsuda-Inoguchi N (2001). Dietary selenium intake of Chinese adult women in the 1990s. Biol Trace Elem Res.

[19] Ge KY (2011). The transition of Chinese dietary guidelines and the food guide pagoda. Asia Pac J Clin Nutr.

[20] Zhang L, Li JG, Liu X, Zhao YF, Li XW, Wen S (2013). Dietary intake of PCDD/fs and dioxin-like PCBs from the Chinese total diet study in 2007. Chemosphere.

[21] Wu YN, Li XW (2015). The fourth China Total diet study.

[22] Zhou PP, Zhao YF, Li JG, Wu GH, Zhang L, Liu Q (2012). Dietary exposure to persistent organochlorine pesticides in 2007 Chinese total diet study. Environ Int.

[23] Tan JA, Zhu WY, Wang WY, Li RB, Hou SF, Wang DC (2002). Selenium in soil and endemic diseases in China. Sci Total Environ.

[24] Cheng YY (2014). A brief introduction to the 2013 revised “Chinese dietary reference intakes (DRIs)”. Acta Nutrimenta Sinica.

[25] Gao J, Liu Y, Huang Y, Lin ZQ, Bañuelos GS, Lam MHW (2011). Daily selenium intake in a moderate selenium deficiency area of Suzhou, China. Food Chem.

[26] Stoffaneller R, Morse NL (2015). A review of dietary selenium intake and selenium status in Europe and the Middle East. Nutrients.

[27] Chun OK, Floegel A, Chung SJ, Chung CE, Song WO, Koo SI (2010). Estimation of antioxidant intakes from diet and supplements in U.S. adults. J Nutr.

[28] Wang ZH, Zhai FY, He Y, Wang HJ, Yu WT, Yu DM (2008). Influence of family income on dietary nutrients intake and dietary structure in China. Journal of Hygiene Research.

[29] Li SM, Banuelos GS, Wu LH, Shi WM (2014). The changing selenium nutritional status of Chinese residents. Nutrients.

[30] Chen LC, Yang FM, Xu J, Hu Y, Hu QH, Zhang YL (2002). Determination of selenium concentration of rice in China and effect of fertilization of selenite and selenate on selenium content of rice. J Agric Food Chem.

